# Transcriptome-Wide Identification of G-to-A RNA Editing in Chronic Social Defeat Stress Mouse Models

**DOI:** 10.3389/fgene.2021.680548

**Published:** 2021-05-19

**Authors:** Ji Tao, Chun-Yan Ren, Zhi-Yuan Wei, Fuquan Zhang, Jinyu Xu, Jian-Huan Chen

**Affiliations:** ^1^Laboratory of Genomic and Precision Medicine, Wuxi School of Medicine, Jiangnan University, Wuxi, China; ^2^Joint Primate Research Center for Chronic Diseases, Institute of Zoology of Guangdong Academy of Science, Jiangnan University, Wuxi, China; ^3^Institute of Zoology, Guangdong Academy of Sciences, Guangzhou, China; ^4^School of Biotechnology, Jiangnan University, Wuxi, China; ^5^Institute of Neuropsychiatry, The Affiliated Brain Hospital of Nanjing Medical University, Nanjing, China; ^6^Department of Emergency Medicine, Wuxi People’s Hospital Affiliated to Nanjing Medical University, Wuxi, China

**Keywords:** RNA editing, social defeat, emotional stress, physical stress, ventral tegmental area, depression, post-traumatic stress disorder

## Abstract

Emerging evidence suggests that RNA editing is associated with stress, neurological diseases, and psychiatric disorders. However, the role of G-to-A RNA editing in chronic social defeat stress (CSDS) remains unclear. We herein identified G-to-A RNA editing and its changes in the ventral tegmental area (VTA), a key region of the brain reward system, in CSDS mouse models under emotional stress (ES) and physiological stress (PS) conditions. Our results revealed 3812 high-confidence G-to-A editing events. Among them, 56 events were significantly downregulated while 23 significantly upregulated in CSDS compared to controls. Moreover, divergent editing patterns were observed between CSDS mice under ES and PS conditions, with 42 and 21 events significantly upregulated in PS and ES, respectively. Interestingly, differential RNA editing was enriched in genes with multiple editing events. Genes differentially edited in CSDS included those genetically associated with mental or neurodevelopmental disorders, especially mood disorders, such as FAT atypical cadherin 1 and solute carrier family 6 member 1. Notably, changes of G-to-A RNA editing were also implicated in ionotropic glutamate receptors, a group of well-known targets of adenosine-to-inosine RNA editing. Such results demonstrate dynamic G-to-A RNA editing changes in the brain of CSDS mouse models, underlining its role as a potential molecular mechanism of CSDS and stress-related diseases.

## Introduction

RNA editing is the change of RNA nucleotide sequence that occurs at the transcriptional level (Tan et al., 2017). Canonical RNA editing in mammals includes adenosine to inosine (A-to-I) editing mediated by the adenosine deaminase acting on RNA (ADAR) protein family and cytidine to uridine (C-to-U) mediated by the Apolipoprotein B mRNA Editing Catalytic Polypeptide-like (APOBEC) protein family (Gu et al., 2012). RNA editing has been found in various organisms, including animals (Tan et al., 2017), plants (Small et al., 2020), and humans (Christofi and Zaravinos, 2019), and is involved in a variety of physiological and pathological processes. Recent studies have suggested that RNA editing significantly changes in response to stress, neurological diseases and psychiatric disorders (Kawahara et al., 2004; Breen et al., 2019; Dick et al., 2019), suggesting that it may be involved in the molecular mechanisms of these pathological processes.

Non-canonical RNA editing refers to change of RNA sequence other than A-to-I or C-to-U canonical RNA editing. Compared to canonical RNA editing, non-canonical RNA editing remains relatively poorly understood. G-to-A RNA editing has been reported in the heterogeneous nuclear ribonucleoprotein K (*HNRNPK*) gene in colorectal cancer and the glutamate ionotropic receptor kainate type subunit 3, *GRIK3*) gene in the fetal human brain (Nutt et al., 1994, 7; Klimek-Tomczak et al., 2006). G-to-A editing in WT1 transcripts (Niavarani et al., 2015) was reported to be mediated by the deaminase activity of APOBEC3A. Nevertheless, the role of G-to-A editing in physiological and pathological processes is largely unclear.

Social defeat stress models are important tools to study post-traumatic stress disorder (PTSD), depression, and other stress-related diseases. Recent studies have shown that social defeat stress could induce changes in RNA editing (Dick et al., 2019). Dick et al. (2019) demonstrated dynamic regulation of A-to-I editing in the prefrontal cortex and basolateral amygdala after chronic social defeat (CSDS) in mice. However, the role of non-canonical G-to-A RNA editing in CSDS remains to be elucidated.

Chronic social defeat stress could be induced in mice under emotional stress (ES) condition by witnessing traumatic events and insulating effects of physical stress (PS) (Warren et al., 2013). Interestingly, both similarities and differences in brain transcript expression profiles were found between CSDS mice under ES and PS conditions in Warren’s study. Therefore, G-to-A RNA editing in CSDS mouse models under different stress conditions is still to be studied. The current study herein identified G-to-A RNA editing from RNA sequencing (RNA-Seq) data of the mouse ventral tegmental area (VTA) at the transcriptome level. Our findings revealed that dynamic changes of G-to-A RNA editing were associated with CSDS, and could also contribute to the difference between CSDS models under ES and PS conditions.

## Materials and Methods

### Collection of RNA-Seq Data

Raw RNA-Seq read data were downloaded from NCBI Gene Expression Omnibus (GEO) database^1^. The samples used for RNA editing event discovery consisted of adult C57BL/6J male mouse brain VTA from CSDS mouse models under ES or PS conditions, and controls (*N* = 3 per group) (GSE36005) (Warren et al., 2013). As described Warren’s study, naïve C57BL/6J mice were assigned to either ES or PS conditions. ES mice were exposed to witnessing the social defeat of a PS mouse attacked by an aggressor CD-1 mouse from a safe adjacent compartment. Another set of VTA samples from 44 adult C57BL/6 mice were analyzed independently to generate a reference dataset, and used to help identify high-confidence RNA editing events (GSE89692) (Peña et al., 2017, 2019).

### Processing of RNA-Seq Data

The raw sequencing data obtained above were first analyzed by FASTQC for quality control. Reads that passed quality control were aligned and mapped to the mouse genome (UCSC mm10) using RNA STAR (version 2.7.0e) (Dobin and Gingeras, 2015), and generating alignment files in BAM format. SamTools (version 1.9) was used to filter the reads by removing optic duplications in the BAM files (Li et al., 2009), and only reads uniquely mapped to the mouse genome were kept. Base quality score recalibration was then performed with the resulting BAM files by using GATK (version 4.1.3) and following the best practices workflows recommended by the documentation (Van der Auwera et al., 2013).

### Identification of High-Confidence G-to-A RNA Editing Events

RNA-Seq data from CSDS and control mice (GSE36005) were used for discovery of RNA editing events. Single nucleotide variants (SNV) were called from the GATK re-calibrated BAM files by using VarScan (version 2.4.3) (Koboldt et al., 2012). The variant calling criteria were set as follows: base quality ≥25, total sequencing depth ≥10, alternative allele depth ≥2 and alternative allele frequency (AAF) ≥1%, and possible false positive SNVs were filtered and removed by using the fpfilter command implemented by VarScan with default parameters. SNVs annotated using the Ensembl Variant Effect Predictor (VEP)^2^ (McLaren et al., 2016). SNVs that met any of the following conditions were further filtered and removed unless annotated as known RNA editing events in the REDIportal V2.0 database^3^ (Mansi et al., 2021): (1) located in simple repeats or homopolymer runs ≥5 nucleotides (nt); (2) located in the mitochondria; (3) located within 6 nt from splice junctions; (4) located within 1 nt from insertion-deletions (INDELs); (5) annotated as known variants in the dbSNP database; or (6) more than 90% of the samples of controls and CSDS had an AAF equal to 100% or between 40 and 60%. RNA-Seq data from another 44 adult mice (GSE89692) were used as an additional dataset to generate a list of reference G-to-A RNA editing events, and help identify high-confidence ones. The same analysis workflow used in the RNA editing event discovery was applied. Only G-to-A SNVs that were located in genic regions, and were detected in at least 2 samples or replicated in the additional set of 44 mouse VTA samples with editing levels ≥1% were kept, which were defined as high-confidence G-to-A RNA editing events.

### Quantification of Gene Expression

FeatureCounts was used to obtain the pseudo-counts of gene expression from the BAM files (Liao et al., 2014), and transcripts read per thousand bases per million mapping (TPM) were then calculated for each gene using EdgeR (version 3.7) (McCarthy et al., 2012).

### Principal Component Analysis

Principal component analysis (PCA) of G-to-A RNA editing events was performed using the function Prcomp in R (version 3.6.3), and visualized using the ggplot2 (version 2.2.1) package.

### Enrichment Analysis of Gene Functions and Pathways

In order to understand the potential biological effects of RNA editing, the analysis of the Gene list enrichment analysis of gene ontology (GO) and kyoko encyclopedia of genes and genomes (KEGG) pathways were conducted using Enrichr^4^ (Kuleshov et al., 2016). Enrichment of with by FDR < 0.05 as the cutoff.

### Statistical Analysis

Levels of RNA editing or gene expression were compared using Student’s *t*-test between groups. *P-*values < 0.05 were considered to be significant. The correlation coefficient (r) between RNA editing levels was calculated using *Spearman* correlation analysis. *Fisher’s* exact test was used to analyze enrichment of differentially edited genes.

## Results

### G-to-A RNA Editing Events Identified in Mouse VTA Transcriptome

**FIGURE 1:**
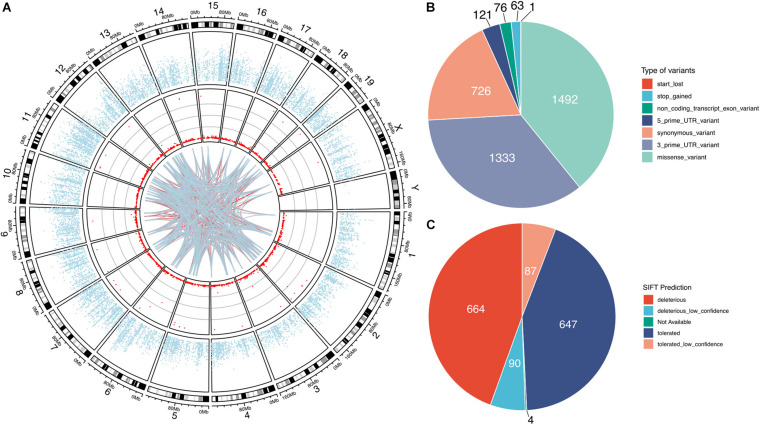
G-to-A RNA editing events identified from adult mouse VTA transcriptome. The red dots denotes the events observed across all chromosomes in the mouse genome **(A)**. The blue dots show mean expression levels of genes. And the lines denote interaction between the G-to-A RNA editing events, with the blue-to-red gradient indicated the correlation co-efficient r. The interaction among the top 100 frequently observed editing events is shown. **(B)** The G-to-A RNA editing events result in various types of mRNA variants. **(C)** About half of these missense events are predicted by SIFT to possibly be deleterious on the encoded proteins.

Our bioinformatic analysis identified a total of 3812 high-confidence *G-to-A* RNA editing events in 2553 genes in the mouse VTA RNA-Seq data (Figure 1 and Supplementary Table 1). These events, with editing levels of these events ranging from 1 to 100%, were widely observed across all chromosomes. These identified high-confidence RNA editing events consisted of various functional categories, including 1492 missense, 1333 3′-untranslated region (UTR), 726 synonymous, 121 5′-UTR, 76 non-coding transcript exons variants, 63 stop-gain, and 1 stop-lost variants (Figure 1B). SIFT predicted 754 (50.5%) of the missense events could have potential impact on the protein function or structure (Figure 1C).

### CSD-Associated G-to-A RNA Editing Events

**FIGURE 2:**
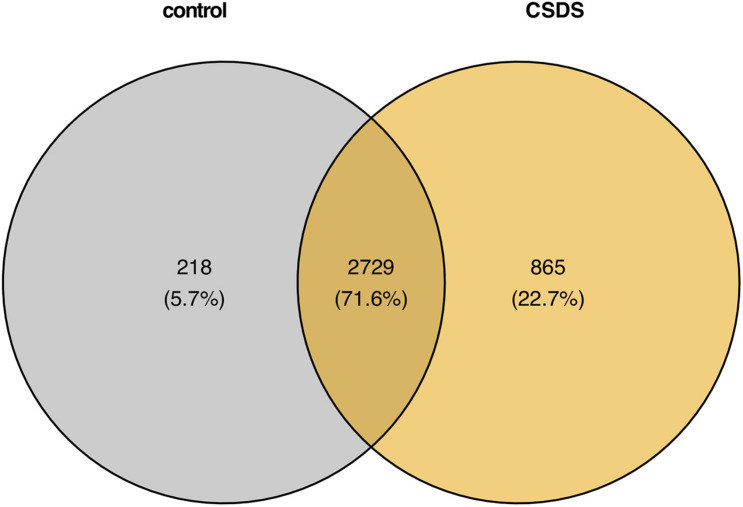
Comparison of genomic locations of G-to-A RNA editing events in controls and CSDS.

**FIGURE 3:**
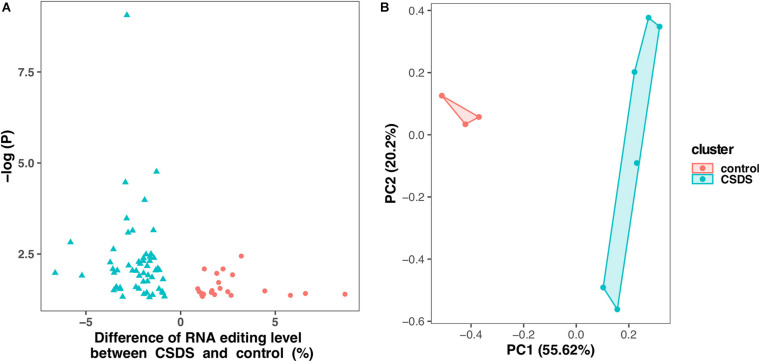
Differential G-to-A RNA editing events between CSDS and controls. **(A)** A total of 79 events are differentially edited between CSDS and controls, including 56 and 23 are upregulated and downregulated, respectively. **(B)** Principle component analysis of the 79 events differentially edited between CSDS and controls.

As shown in Figure 2, comparison of G-to-A RNA editing between CSDS and controls revealed that, 2729 (71.6%) were shared by both CSDS and controls, 865 (20.8%) were observed only in CSDS but not in controls, and 871 (24.2%) only in controls but not in CSDS. *T*-test identified a total of 79 G-to-A RNA editing events with significantly different editing levels, among which 56 were downregulated and 23 were upregulated in CSDS compared to controls, respectively (Figure 3A). These CSDS-associated events consisted of 36 in 3′-UTR, 2 in 5′-UTR, 4 in non-coding transcript exons, and 20 in coding regions including 20 missense and 17 synonymous variants, with no significant expression changes in most of the genes with differential editing (Supplementary Table 2). 22 of the genes with differential G-to-A editing in CSDS had been reported to be associated with psychiatric diseases, and another five of them were associated with other neurological diseases. PCA with these 79 differential editing events showed that CSDS samples clustered separately from controls, with 55.62% contribution from PC1 to the total variance (Figure 3B).

**FIGURE 4:**
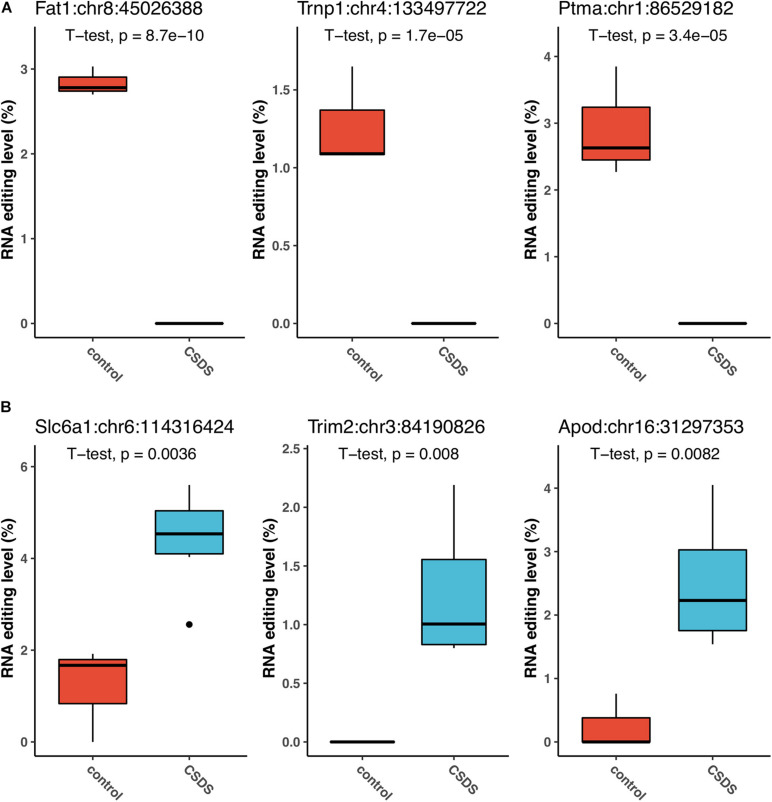
The events that are the most significantly associated with CSDS. The top three upregulated events **(A)**, and top three downregulated events **(B)** in CSDS compared to controls are shown.

The three G-to-A RNA editing events that were the most significantly downregulated in CSDS compared to controls were observed in FAT atypical cadherin 1 (*Fat1*:chr8:45026388, *P* = 8.7 × 10^–10^), TMF1 regulated nuclear protein 1 (*Trnp1*:chr4:133497722, *P* = 1.7 × 10^–5^), and prothymosin alpha (*Ptma*:chr1:86529182, *P* = 3.4 × 10^–5^) (Figure 4A). Notably, the *Fat1* RNA editing at *Fat1*:chr8:45026388, which produced a missense change p.V280M (Gtg- > Atg), and another *Fat1* missense event at *Fat1*:chr8:45025105 (aGc/aAc, p.S2373N) were observed in controls only. Both of the two *Fat1* G-to-A changes were predicted to be deleterious to the protein function (Supplementary Table 2). In contrast, the top three G-to-A RNA editing events upregulated in CSDS were found in solute carrier family 6 member 1 (*Slc6a1*:chr6:114316424, *P* = 0.0036), tripartite Motif Containing 2 (Trim2:chr3:84190826, *P* = 0.008), and apolipoprotein D (Apod:chr16:31297353, *P* = 0.0082) (Figure 4B). In addition, our results also implicate that glutamate ionotropic receptor NMDA type subunit 2b (*Grin2b*) and subunit associated protein 1 (*Grina*) could be the two most evident glutamate receptors subjected to G-to-A RNA editing in CSDS (*Grin2b*:chr6:135720485, *P* = 0.0178; and *Grina*:chr15:76248349, *P* = 0.0287, respectively) (Supplementary Table 2). These RNA editing events, either differentially edited or exclusively detected in either controls or CSDS, suggested that changes of G-to-A RNA editing could be associated with CSDS.

### Functional Enrichment in CSDS-Associated G-to-A RNA Editing

**FIGURE 5:**
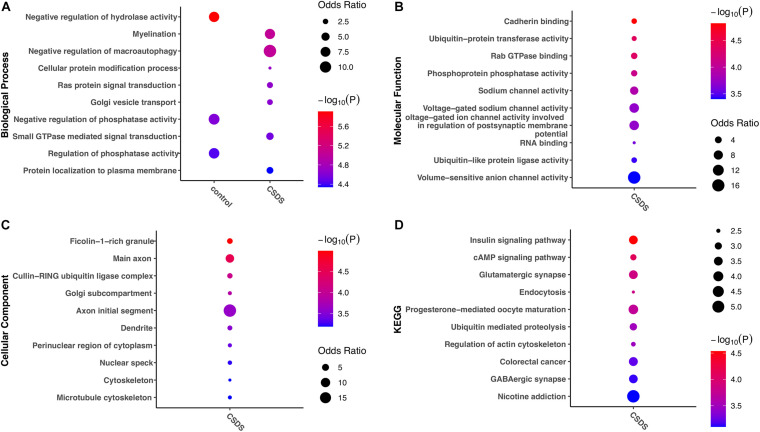
Gene ontology and KEGG pathways erniched in gene with CSDS-associated G-to-A RNA editing. The items with the most significant *P*-values are shown for **(A)** biological processes, **(B)** molecular functions, and **(C)** cellular components, as well as **(D)** KEGG pathways enriched in either CSDS or controls. CSDS, chronic social defeat stress.

To help understand the biological impact of G-to-A RNA editing in CSDS, enrichment analysis of genes with RNA editing enriched in either controls or CSDS was further compared. Intriguingly, the results in Figure 5 showed stronger functional enrichment in CSDS than in controls. GO analysis showed that biological processes enriched in CSDS included myelination, negative regulation of macroautophagy, cellular protein modification, Ras protein signal transduction, Golgi vesicle transport, small GTPase mediated signal transduction, and protein localization to plasma membrane (Figure 5A). The molecular functions enriched in CSDS were mainly related to cadherin binding, ubiquitin-related enzyme activity, sodium channel activity, Rab GTPase binding, and RNA binding (Figure 5B). The cellular components enriched in CSDS were mainly related to ficolin-1-rich granule, cullin-RING ubiquitin ligase complex, Golgi subcompartment and neuron-specific components such as main axon (Figure 5C). The KEGG pathways enriched in CSDS were mainly involved in insulin signaling, cAMP signaling, endocytosis, ubiquitin mediated proteolysis, glutamatergic and GABAergic synapse, regulation of actin cytoskeleton as well as neurological diseases such as nicotine addiction (Figure 5D).

### Divergent G-to-A RNA Editing Patterns Between Different CSDS Models

Although both ES and PS mice were reported to exhibit similar defects of depression- and anxiety-like behavior, ES mice experienced CSDS by only witnessing emotional events without direct physical interaction. Previously Warren’s study indicated possible differences in transcript expression between the two CSDS models. We thus went on to compare the G-to-A RNA editing patterns between the two CSDS paradigms.

**FIGURE 6:**
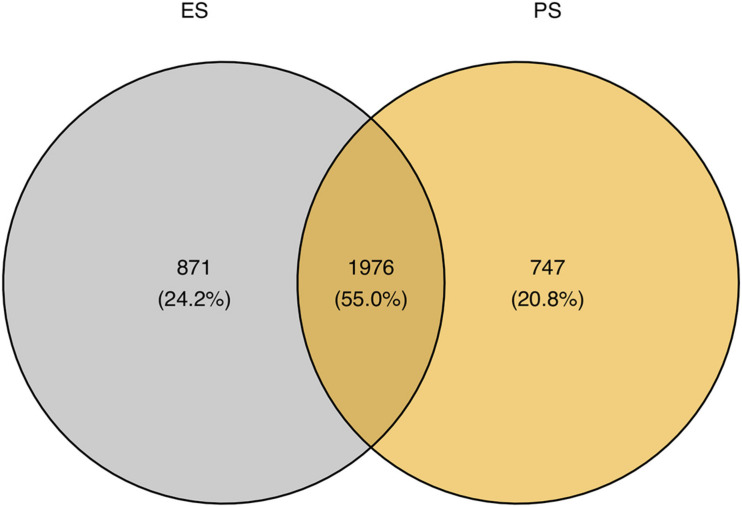
Comparison of genomic locations of G-to-A RNA editing events between ES and PS.

**FIGURE 7:**
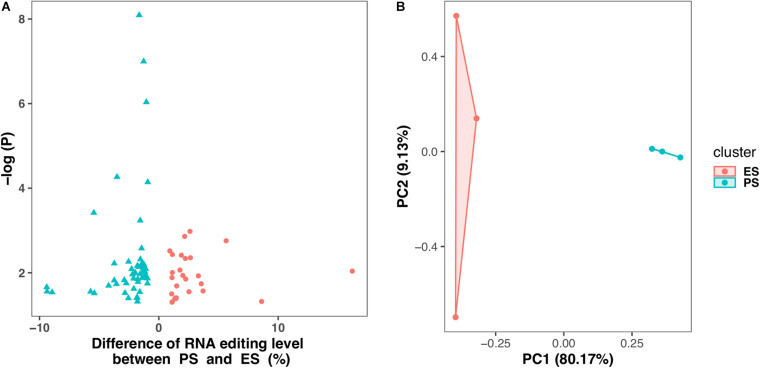
Differential G-to-A RNA editing events between PS and ES. **(A)** 63 events showed differential editing levels between CSDS and controls, including 42 and 21 are upregulated in PS and ES, respectively. **(B)** Principle component analysis of these Differential G-to-A RNA editing between PS and ES.

**FIGURE 8:**
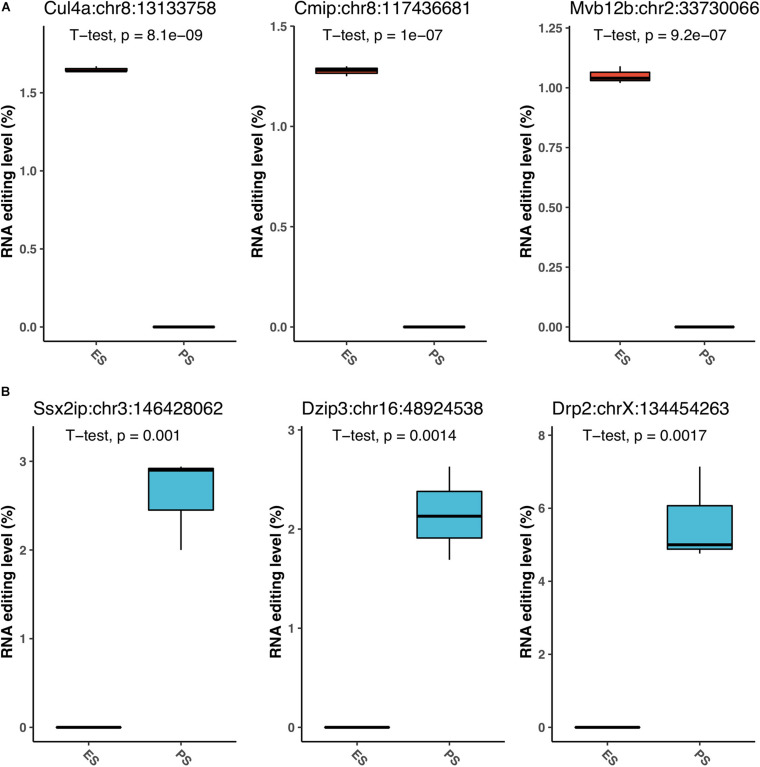
The top G-to-A RNA editing with significant difference between PS and ES. The top three upregulated events in ES **(A)**, and top three downregulated events **(B)** in PS are shown.

Figure 6 showed that among these RNA editing events, 1976 (55.0%) were shared by both ES and PS, 787 (20.8%) were observed only in PS but not in ES, and 871 (24.2%) only in ES but not in PS. Furthermore, Student’s *t*-test identified 63 events with significantly different editing levels between ES and PS, among which 42 were upregulated in ES and 21 were upregulated in PS (Figure 7A and Supplementary Table 3). Five of the genes differentially edited between ES and PS were associated with psychiatric diseases, and another six of them were associated with other neurological diseases. As shown in Figure 8, the top three events that significantly upregulated in ES were found in cullin 4A (*Cul4a*:chr8:13133758, *P* = 8.09 × 10^–9^), c-Maf inducing protein (*Cmip*:chr8:117436681, *P* = 1.01 × 10^–7^), and multivesicular body subunit 12b (*Mvb12b*:chr2:33730066, *P* = 9.24 × 10^–7^, respectively). The top three events that significantly upregulated in PS were found in SSX family member 2 interacting protein (*Ssx2ip*:chr3:146428062, *P* = 0.001), DAZ interacting zinc finger protein 3 (*Dzip3*: chr16:48924538, *P* = 0.001), and dystrophin related protein 2 (*Drp2*:chrX:134454263, *P* = 0.001). PCA with these 63 events showed that ES cluster separately from PS, with higher intra-group variance in ES, with PC1 contributing 80.2% to the total variance, (Figure 7B). These events, either enriched in ES or PS, indicated that divergent patterns of G-to-A RNA editing might contribute to the underlying different mechanisms in the two CSDS models.

### Functional Divergence of G-to-A RNA Editing Enriched in ES or PS

**FIGURE 9:**
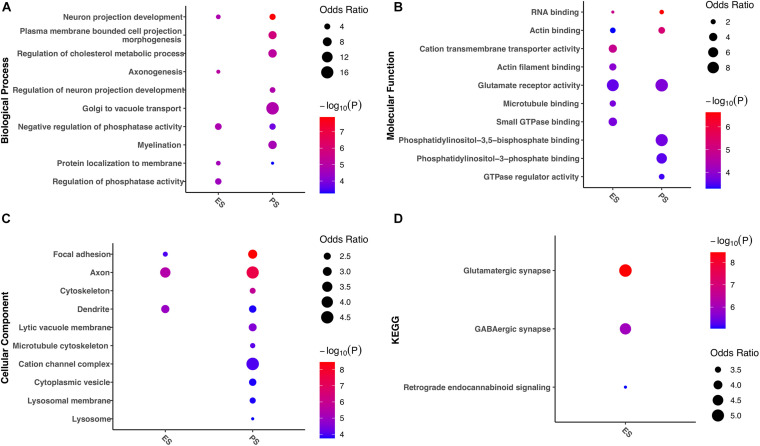
Gene ontology and KEGG pathways associated with divergent G-to-A RNA editing patterns between PS and ES. The items with the most significant *P*-values are shown for **(A)** biological processes, **(B)** molecular functions, and **(C)** cellular components, as well as **(D)** KEGG pathways. ES, emotional stress; PS: physical stress.

Enrichment analyses of gene functions and pathways were further evaluated in genes with G-to-A RNA editing enriched in either ES or PS (Figure 9). The results demonstrated that a number of these gene functions were found in both CSDS models. Biological processes such as neuron projection development, negative regulation of phosphatase activity, and protein localization to membrane, molecular functions such as RNA binding and actin binding, glutamate receptor activity, and cellular components such as focal adhesion, axon, and dendrite were enriched in both models. Nevertheless, more gene functions and pathways were significantly enriched in either of the two CSDS models but not the other. For example, biological processes related to cholesterol metabolism, neuron projection development, Golgi to vacuole transport and myelination were enriched in PS. In contrast, biological processes related to axonogenesis, and protein localization to membrane, and pathways related to GABAergic synapse and Retrograde endocannabinoid signaling were specifically enriched in ES.

### Enrichment of Differential G-to-A RNA Editing in Genes With Multiple Editing Events

It was noted that a large proportion of genes in the mouse VTA RNA-Seq were observed to contain multiple G-to-A RNA editing events. The top ten genes with the largest counts of editing events are shown in Table 1. The microtubule associated protein 1b (*Map1b*) and HECT, UBA And WWE domain containing E3 ubiquitin protein ligase 1 (*Huwe1*) genes, were found to be the top two genes with the largest counts of editing events. Moreover, differential G-to-A RNA editing was also observed in the two genes. As illustrated in Table 2, *Fisher’s* exact test implicated that differential G-to-A RNA editing between control and CSDS or between PS and ES were dramatically enriched in genes with more than one editing event (OR = 2.6, 95% CI = 1.5–4.5, *P* = 2.26 × 10^–4^ and OR = 2.6, 95% CI = 1.6–4.2, *P* = 5.08 × 10^–5^).

**TABLE 1:** Top ten genes with the largest counts of G-to-A RNA editing events.

**No.**	**Gene**	**Number of detected editing events**	**With significantly different G-to-A RNA editing levels**	
			**Between control and CSDS**	**Between PS and ES**
1	*Map1b*	14	No	Yes
2	*Huwe1*	9	No	Yes
3	*Gm20721*	8	No	No
4	*Lrp1*	8	No	No
5	*Mgat3*	7	No	No
6	*Shisal1*	7	No	No
7	*Sptbn1*	7	No	No
8	*Srrm2*	7	No	Yes
9	*Adarb1*	6	No	No
10	*Ank2*	6	No	No
				

**TABLE 2:** Enrichment of differential G-to-A RNA editing in genes with multiple editing events.

**Genes with significant different levels**		**Counts of editing events**		**OR**	P*
		**Counts = 1**	**Counts > 1**	**(95% CI)**	
Between PS and ES	No	1739	752	2.6	2.26 × 10^–4^
	Yes	29	33	(1.5–4.5)	
Between control and CSDS	No	1731	744	2.6	5.08 × 10^–5^
	Yes	37	41	(1.6–4.2)	

*P-values are calculated using *Fisher’s* exact test.

## Discussion

The rodent model of CSDS has been widely used in the study of stress-related diseases such as major depression and PTSD, providing an important and useful tool for the understanding of these diseases. The current study for the first time systematically investigated G-to-A RNA editing in the brain VTA in CSDS mice at the transcriptome-level, and demonstrated dynamic changes of G-to-A RNA editing associated with CSDS as well as divergent editing patterns found under ES or PS conditions.

Ventral tegmental area is a key region of the dopaminergic brain reward system, and its role in mood and anxiety disorders has been implicated by emerging evidence (Baik, 2020). Therefore, our results demonstrate that widespread G-to-A RNA editing is found in the mouse VTA transcriptome, indicating its potentially important biological functions in the brain. Nevertheless, the actual biological functions of G-to-A RNA editing, and how it is related to neurological and mental disorders remain largely unclear. By far a number of targets have been reported for G-to-A RNA editing, such as *HNRNPK* in colorectal cancer (Klimek-Tomczak et al., 2006) and *WT1* in cord blood samples (Niavarani et al., 2015). For neural tissue and central nerve system, G-to-A editing was reported in *GRIK3* in the fetal human brain (Nutt et al., 1994). Moreover, G-to-A editing led to a missense variant p.R441H in TPH2 (Grohmann et al., 2010) with decreased its enzyme activity, which had been associated with major depression disorder (Zhang et al., 2005). In the current study, multiple G-to-A editing events in the 3′-UTR of mouse *Grik3* were observed. In addition, a high proportion of missense and stop-gain events were detected in genes with high expression levels in VTA. It is also worth noting that coding variants accounted for a high proportion (59.9%) of the RNA editing variants found, while missense, stop-gain and stop-lost variants accounted for another 68.2% of the coding variants. This may imply that G-to-A RNA editing could have a potential impact on the sequences of protein expressed in VTA. Such findings thus underscore the potential role of G-to-A editing in the brain.

Recently, RNA editing has been reported to be involved in CSDS. For example, by using target sequencing, Dick et al. (2019) reported dynamic regulation of A-to-I editing in 5-Hydroxytryptamine Receptor 2C (*Htr2c*) in CSDS mice. In line with such existing evidence, our results revealed changes of G-to-A editing events in CSDS mice, suggesting that CSDS could have dramatic effects on G-to-A editing in the brain. Moreover, divergent G-to-A editing patterns contributed to the difference between CSDS paradigms induced under ES and PS conditions. As a result, gene list enrichment further suggested that such changes may lead to altered gene functions or pathways in the brain.

It was noteworthy that a number of the genes with differential G-to-A editing in CSDS had been reported to be associated with neurological or psychiatric diseases, or functions as key components in neurons (Supplementary Table 2). In the current study, the most significant difference between CSDS and controls was observed in *Fat1*. It encodes a member of the cadherin superfamily. Two missense events in *Fat1* were exclusive found in controls but not CSDS (Figure 4A and Supplementary Table 2), which might affect its protein function or structure., and its human ortholog *FAT1* is associated with nicotine dependence and major depression (Blair et al., 2006). Likewise, the gene with the most significantly upregulated G-to-A RNA editing was found of *Slc6a1*, which encodes for the GABA transporter protein type 1 (also known as *Gat1*). The Slc6a1 transporter removes GABA, the primary inhibitory neurotransmitter in the CNS, from the synaptic cleft, and maintains low extracellular levels of GABA. Mutations of *SLC6A1* were reported in a variety of neurological and psychiatric disorders, myoclonic atonic epilepsy, intellectual disability (Johannesen et al., 2018, 1), autism (Wang et al., 2020), as well as schizophrenia (Rees et al., 2020). Association of *Slc6a1* with mood disorders had also been implicated. *Slc6a1* deficient mice demonstrated reduced depression and anxiety-like behaviors (Liu et al., 2007; Gong et al., 2015). Moreover, genetic polymorphisms of *SLC6A1* were reported to be associated with anxiety (Thoeringer et al., 2009). In line with such existing evidence, a trend of increased expression of *Slc6a1* was found in CSDS mice with marginal significance (*P* = 0.087) together with increased G-to-A RNA editing in the in the 3′-UTR. Such findings, taken together, pointed to a potential effect of G-to-A RNA editing on *Slc6a1* expression and GABAergic system in CSDS.

In particular, our findings re-emphasize the potential importance of RNA editing glutamate receptors, which are linked to fast excitatory neurotransmission in the CNS. Ionotropic glutamate receptors consist of three groups according to their specific agonists, includingα-amino-3-hydroxy-5-methyl-4-isoxazolepropionic acid (AMPA) receptors, N-methyl-D-aspartate (NMDA) receptors, and kainate receptors. By far, Non-NMDA glutamate receptors have been known to be important targets for A-to-I RNA editing. Interestingly, our results implicate that NMDA including glutamate ionotropic receptor NMDA type subunit 2b (*Grin2b*) and glutamate ionotropic receptor NMDA type subunit associated protein 1 (*Grina*) could be the two most significant glutamate ionotropic receptors subjected to G-to-A RNA editing in CSDS. In addition, G-to-A RNA editing was also found in kainate receptors and AMPA receptors (Supplementary Table 1). GO analysis showed that the function of glutamate receptor-related gene functions was enriched in genes with G-to-A RNA editing in CSDS, suggesting that glutamate receptor-related genes may also be important targets of G-to-A RNA editing, which could be related to stress response.

In addition, divergent RNA-editing patterns were observed between ES and PS in the current study (Supplementary Table 3). The most significantly differential editing was found in *Cul4a*. It was noteworthy that a SNP in *CUL4A* was found to be associated with bipolar disorder in a genome-wide association study (GWAS) (Stahl et al., 2019). Moreover, a recent analysis revealed *CUL4A* as one of the 112 hub genes in the complex network of GWAS-identified genes in bipolar disorder (Xie et al., 2017). Therefore, the differential G-to-A RNA editing in *Cul4a* were probably in line with a potential role of the gene in emotional stress and mood disorders.

G-to-A RNA editing was not evenly distributed among expressed genes in the transcriptome. A number of genes were found to contain more than one editing event, suggesting that these genes might be editing hotspots. Moreover, genes with multiple editing events showed a significantly higher possibility to be differentially edited in CSDS compared to controls. These genes are mostly involved in development and function of the nervous system, or associated with neurological and mental disorders (Supplementary Tables 2, 3). For example, the *Map1b* gene encodes a protein member of the microtubule-associated protein family involved in essential microtubule assembly in neurogenesis. Loss-of-function mutations of *MAP1B* were reported in patients with intellectual disability (Walters et al., 2018). Another example was *Huwe1*, which encodes an E3 ubiquitin ligase, and plays a key role in cerebral cortex neurogenesis (Zhao et al., 2009; D’Arca et al., 2010). Mutations in this gene were reported in including both non-syndromic and syndromic forms of X-linked intellectual disability (Moortgat et al., 2018; Muthusamy et al., 2020).

In conclusion, the current study identified high-confidence G-to-A RNA editing in the VTA transcriptome of adult male mouse brains, and revealed dynamic changes in different CSDS models. With a number of stress/mood disorder-related genes revealed to be involved in altered G-to-A RNA editing, especially those with multiple editing events, our findings thus provide new insight into the understanding of the role played by brain G-to-A RNA editing in CSDS and its related diseases.

## Data Availability Statement

The original contributions presented in the study are included in the article/Supplementary Material, further inquiries can be directed to the corresponding authors.

## Ethics Statement

The animal study was reviewed and approved by the Animal Ethics Committee of Jiangnan University.

## Author Contributions

JT and C-YR performed the bioinformatic analysis. Z-YW improved the data analysis pipeline. JX and J-HC conceived the project and planned the experiments. FZ participated in the discussion. All authors contributed to the final manuscript.

## Conflict of Interest

The authors declare that the research was conducted in the absence of any commercial or financial relationships that could be construed as a potential conflict of interest.

## Footnotes

1 https://www.ncbi.nlm.nih.gov/geo/

2 https://www.ensembl.org/vep

3 http://srv00.recas.ba.infn.it/atlas/index.html

4 https://maayanlab.cloud/Enrichr/
